# Social support and avoidance mediate positive and negative effects of emotion recognition ability on mental health in medical students

**DOI:** 10.1038/s41598-025-02025-8

**Published:** 2025-05-15

**Authors:** Nils R. Sommer, Valerie Carrard, Céline Bourquin, Alexandre Berney, Katja Schlegel

**Affiliations:** 1 Institute of Psychology, University of Bern, Bern, Switzerland; 2 Psychiatric Liaison Service, Lausanne University Hospital (CHUV) and University of Lausanne, Lausanne, Switzerland

**Keywords:** Emotion recognition ability, Emotional intelligence, Social support, Mental health, Human behaviour, Health occupations

## Abstract

The ability to recognize others’ feelings from nonverbal expressions, known as emotion recognition ability (ERA), is considered a crucial socio-emotional competence that may enhance both intra- and interpersonal functioning in healthcare professionals. However, evidence for its association with mental health is mixed. The present longitudinal study examined whether medical students with higher ERA scores report better mental health over one year and whether this effect is mediated by a higher perceived availability of social support. Longitudinal mediation analyses were conducted with data from 986 medical students in Switzerland who completed questionnaires at two time points, one year apart. While ERA at T1 was not directly associated with mental health issues and burnout at T2, it predicted greater social support availability at T2, which in turn predicted fewer mental health issues and lower burnout. Exploratory analyses revealed that although ERA increased social support, it also predicted higher habitual avoidance coping, which was negatively related to mental health. Overall, this study sheds light on both positive and negative indirect pathways through which ERA may affect mental health in future healthcare professionals. These insights highlight the need for careful consideration of ERA intervention studies, addressing both positive and negative influences on mental health.

## Introduction

Emotion recognition ability (ERA), i.e., the ability to correctly assess others’ thoughts and feelings from nonverbals cues, is a crucial skill for psychosocial functioning and has been linked to various positive social interaction outcomes. For instance, people who are more accurate at reading others’ emotions have been judged as more socially skilled^1,2^, cooperative and likable^3^, and reported higher relationship quality^1,4^. Such interpersonal benefits of high ERA have also been reported in the field of healthcare, where high-stakes interactions and empathy are central to quality patient care^5^ and higher ERA may enable healthcare workers to gather relevant social information, adapt to patient needs, and engage effectively with colleagues^6,7^. For example, healthcare professionals with higher ERA received higher ratings of empathic communication and patient-centered behavior^2,8^ and have more satisfied clients or patients^9,10^.

While the interpersonal benefits of high ERA are well-documented, evidence on its potential benefits for one’s own well-being and mental health is limited. Although this connection seems plausible, given that positive social interactions and relationships—linked to higher ERA—are essential to mental health^11^, studies on the ERA-well-being relationship in non-clinical populations are uncommon and tend to find limited direct effects, often using cross-sectional designs^12^. Notably, only one previous study has examined this link among healthcare professionals, who face a particularly high risk of mental health challenges compared to other occupations^13,14^. This study was a cross-sectional analysis of a subset of the dataset used in the present study^15^, and found that self-reported empathy, but not performance-based ERA, correlated with mental health issues in medical students^16^. The present study therefore seeks to extend our understanding of the link between ERA and mental health in future healthcare professionals by investigating whether ERA constitutes a protective factor for mental health in a large sample of medical students over a period of one year. Furthermore, the study is the first to examine whether the perceived availability of social resources may explain such a link.

ERA is seen as a key emotional competence within ability emotional intelligence (ability EI) frameworks, particularly in Mayer and Salovey’s four-branch EI model^7^. This model suggests that accurately perceiving emotions in others through nonverbal cues enhances the understanding and management of emotions in oneself and others in social contexts. ERA also constitutes the best studied aspect in the broader realm of interpersonal accuracy, which reflects “the ability to accurately assess others’ emotions, personality, intentions, motives, and thoughts”^6^. Like in the ability EI field, interpersonal accuracy research posits that higher ERA is crucial for understanding social interaction partners and then adapting one’s behavior accordingly to achieve social goals^6^.

In both conceptualizations, ERA is measured using performance-based tests that involve identifying emotions from pictures or videos of human faces or bodies displaying nonverbal emotion expressions^17^. Importantly, the measurement approach in ERA and ability EI research (consisting of items with correct and incorrect responses, akin to measures of cognitive intelligence) is distinct from self-report questionnaires in which participants rate their socio-emotional skills, including how well they think they can read others’ emotions (e.g., “trait” EI questionnaires^18^). Ability and trait EI measures are moderately correlated at best^1,19–21^ and tap into different constructs, with ERA/ ability EI representing a cognitive ability and trait EI overlapping strongly with personality traits like self-esteem^19^.

Although ERA is considered beneficial for interpersonal outcomes, evidence on its association with mental health and subjective well-being is mixed. ERA is impaired in a wide range of mental disorders (e.g., major depression^22,23^) and is discussed to play an important role in their onset and maintenance^24^. However, a small meta-analysis of cross-sectional studies^12^ and a recent daily diary study^25^ found no significant relationship between ERA and subjective well-being in typically-functioning adults. For instance, individuals with higher ERA did not experience more positive affect or report higher life satisfaction^4,12,25^. Nevertheless, another meta-analysis of non-clinical samples linked higher ERA to lower depressive symptoms^1^, and a study conducted during a COVID-19 lockdown found that while individuals with higher ERA did not report more positive affect, they reported less negative affect and felt less burdened^26^. This suggests that being good at reading other people’s emotions does not make people happier, but that it could protect them from developing mental health issues related to depression, anxiety, or burnout by buffering the negative effects of stress. Schlegel^12^ pointed out that the mixed effects found in previous research might be due to parallel processes that cancel each other out^27^. On the one hand, high ERA may foster the quality of social relationships^1^, which could function as a stress buffer^28^ and explain the findings detailed above. On the other hand, according to the “hypersensitivity hypothesis”^29^, high ERA may involve a higher attunement and reactivity to negative emotional situations and stress^12,30–32^, which would be detrimental to mental health as explained by various stress-vulnerability models^33^.

The availability of social support appears to be an important mediator in this regard. Individuals with higher ERA are often seen as more likable and popular and report better social relationships^1,3,4,34^. They might thus be better at building meaningful and reliable social networks that can provide more opportunities to receive emotional and practical support during stressful work-related or other challenges^35^. This may in turn help preventing negative effects on well-being and mental health, in line with previous research^11^. For example, Cohen and Wills^28^ theorized that perceived social support leads to an appraisal of enhanced capacities to deal with stress, thereby buffering its negative effects on mental health. This is supported by meta-analyses on the benefits of perceived social support for the prevention of burnout in students^36^ and for mental health in samples of first responders, e.g., police officers or emergency medical professionals that experience heightened levels of stress^37^. Thus, it can be hypothesized that higher ERA predicts higher perceived availability of social support, which in turn may lead to fewer mental health issues like depression, anxiety, or burnout.

To our knowledge, this hypothesis has not yet been directly tested using mediation analysis and/ or longitudinal designs. However, supporting this idea, a cross-sectional study found that higher ability EI, assessed with an instrument including ERA items, was linked to better mental health, fully mediated by social support^38^. Interestingly, in another study by Zeidner and colleagues^39^ no direct correlation between overall ability EI or ERA and mental health was found, but all three variables were related to social support (a mediation was not formally tested). These findings illustrate that ERA may predict mental health through perceived social support even without an overall effect. Of note, there are some studies in which subjective well-being and mental health were predicted by self-reported trait EI, and this relationship was mediated by social support^40,41^. However, given that trait EI questionnaires (assessing how people see themselves and what they *typically do*) are largely unrelated to performance-based ability EI tests (assessing what people *can do*), such findings cannot readily be transferred to people’s emotional abilities.

The present study thus closes an important gap by examining whether medical students’ ability to accurately decode emotional cues (ERA) predicts lower mental health issues and burnout over a one-year time period (H1), and whether perceived availability of social support mediates this relationship (H2) in a large sample of medical students. Specifically, we hypothesize that higher ERA predicts greater perceived availability of social support (H2a) and that greater perceived availability of social support is associated with lower mental health issues and burnout over time (H2b). Medical students show more depression and anxiety symptoms compared to the general population and burnout levels increase during medical school^14^. Thus, they are a high-stress group where ERA and social support may protect mental health. Moreover, it is important to address mental health issues and burnout during undergraduate medical education since these issues can impair students’ ability to learn essential clinical skills and could persist into their medical practice if left untreated.

## Method

### Study design and participants

This is a secondary analysis of data collected within the ETMED-L project, a four-year longitudinal study investigating medical students’ interpersonal competence and mental health with an open-cohort design^15^. From 2021 to 2024, all medical students from curriculum year 1 to 6 registered in the University of Lausanne (Switzerland), except external academic exchange students, were invited to fill in a 60 min online questionnaire annually. They received CHF50 (~USD50) for each completed yearly questionnaire. At each time point, participants completed an ERA performance test, questionnaires on perceived availability of social support, stress, anxiety, depression, and burnout symptoms, and other measures that were not used in the present analysis (e.g., empathy)^42^. All instructions and instruments were presented in French. The ETMED-L project was approved by the Human Research Ethics Committee of the Canton de Vaud (protocol number 2020–02474), all procedures were performed in accordance with the declaration of Helsinki, and all participants gave written informed consent. An overarching power analysis for the whole project was conducted using Monte Carlo simulation, which suggested a sample size of 525 participants (35% response rate in 1500 potential participants) to find small longitudinal effects^15^.

After excluding 110 participants due to failed attention checks or technical issues, data from 1881 medical students were available. From this sample, 871 students who did not participate for at least two consecutive years were excluded, along with 16 students missing ERA or social support data and 8 students with implausibly low ERA scores (more than 3 standard deviations below the mean). The final sample included 986 medical students with complete data on all variables. Mean age of the final sample was 21.69 years (range: 16–49); see further sample descriptives in (Table 1). For this study, participants’ ERA scores and demographics were taken from their first participation year (T1), and all other variables from the assessment one year later (T2). For students who participated more than two consecutive years, data from their two first participations were used as T1 and T2.

**Table 1:** Sample descriptives at first participation (T1).

Variables	Categories	N	%
Gender identification	Male	310	31.44
	Female	670	67.95
	Non-binary	6	0.61
Curriculum year	1	289	29.31
	2	217	22.01
	3	201	20.39
	4	147	14.91
	5	130	13.18
	6	2	0.20
Number of participations	2	388	39.35
	3	360	36.51
	4	238	24.14
Mother’s education	Mandatory school	227	23.02
	High School	62	6.29
	College	141	14.30
	University	522	52.94
	No answer	34	3.45
Father’s education	Mandatory school	224	22.72
	High school	86	8.72
	College	208	21.10
	University	438	44.42
	No answer	30	3.04
Mother tongue	French	805	81.64
	Italian	50	5.07
	English	30	3.04
	Portuguese	22	2.24
	German	22	2.23
	Spanish	9	0.9
	Other	48	4.88
Family origin	Switzerland	758	76.88
	Neighboring countries	109	11.05
	Other European countries	54	5.46
	Non-European countries	49	4.99
	Unknown	16	1.62

### Measures

#### Emotion recognition ability

ERA at T1 was assessed with the Geneva Emotion Recognition Test Short form (GERT-S)^43^, a performance-based test with 42 short video clips featuring ten actors expressing one of 14 emotions through a standard pseudo-linguistic sentence. Participants selected the expressed emotion after each clip, resulting in a total score reflecting the percentage of correctly recognized emotions. The video stimuli contain multiple modalities (face, voice, and body) and a wide range of emotions. A meta-analysis showed that the GERT had the highest average reliability and correlation with other ERA tests^44^, and several studies support its construct and predictive validity^44,45^.

#### Social support

Two items adapted from the Swiss Household Panel study^46^ measured perceived availability of emotional and practical social support at T2: “To what extent do you have someone in your circle who can be available in case of need and show understanding, by talking with you, for example?” and “If necessary, to what extent can someone in your circle provide you with practical help, that is, concrete help or useful advice?”. Both items were rated on a Likert-scale from 0 (“not at all”) to 10 (“a great deal”).

#### Mental health issues

Depression symptoms at T2 were assessed with the validated French version of the Center for Epidemiological Studies-Depression (CES-D) measuring the experience of symptoms associated with depression over the past week^47,48^. Anxiety symptoms were assessed at T2 with the validated French version^49^ of the trait subscale of the State-Trait Anxiety Inventory (STAI), which measures the level of anxiety that participants “generally feel”^50^. Stress at T2 was measured with a single item assessing general stress level: “Globally, how would you evaluate your current stress level?” on a Likert scale from 1 ‘none’ to 10 ‘extreme’.

#### Burnout

Burnout was measured at T2 with the validated French version^51^ of the Maslach Burnout Inventory Student-Survey (MBI-SS)^52^, which evaluates the three dimensions of Emotional Exhaustion, Cynicism, and Academic Efficacy (reversed dimension).

### Statistical analyses

Two mediation models were estimated: one testing the mediation role of social support in the link between ERA and mental health issues and the other testing the mediation role of perceived social support in the link between ERA and burnout. Mental health issues (including depression symptoms, anxiety symptoms, and stress) and burnout (emotional exhaustion, cynicism, and academic efficacy) were estimated as latent constructs loading on their specific indicators to handle multidimensionality and measurement errors. This approach was chosen due to theoretical (burnout as a distinct construct measured with a single questionnaire) and empirical considerations (high intercorrelations within burnout and mental health scales but lower intercorrelations between these constructs; see Table 2). Social support was also estimated as a latent construct indicated by emotional and practical support items. Students’ gender (male = 1 vs. female/non-binary = 0) and age at T1 were included as control variables. Models were fitted with robust standard errors to account for potential deviations from normal distribution. Model fit was evaluated using the Comparative Fit Index (CFI > 0.95), Root Mean Square Error of Approximation (RMSEA < 0.06), and Standardized Root Mean Square Residual (SRMR < 0.08)^53^. The mediation role of social support was tested using the Monte Carlo Test for Mediation by Zhao and colleagues^54^. All analyses were conducted in STATA version 18^55^.

**Table 2:** Bivariate correlations. Gender identification: Female/nonbinary = 0, male = 1.

Variables	(1)	(2)	(3)	(4)	(5)	(6)	(7)	(8)	(9)	(10)
(1) Emotion recognition ability	1.00									
(2) Emotional support	0.12	1.00								
(3) Practical support	0.04	0.61	1.00							
(4) Depression symptoms	0.01	−0.29	−0.29	1.00						
(5) Anxiety symptoms	0.05	−0.24	−0.25	0.75	1.00					
(6) Stress	0.03	−0.15	−0.14	0.60	0.56	1.00				
(7) Burnout: emotional exhaustion	0.03	−0.18	−0.2	0.62	0.57	0.51	1.00			
(8) Burnout: cynicism	0.07	−0.17	−0.15	0.44	0.35	0.23	0.47	1.00		
(9) Burnout: academic efficacy	−0.04	0.21	0.25	−0.47	−0.48	−0.32	-0.46	−0.50	1.00	
(10) Gender identification	−0.15	−0.09	−0.07	−0.23	−0.26	−0.19	-0.19	−0.06	0.05	1.00
(11) Age	0.03	0.01	−0.01	−0.07	−0.04	−0.05	-0.1	0.07	0.01	0.08

## Results

### Mediation models

**Fig. 1:**
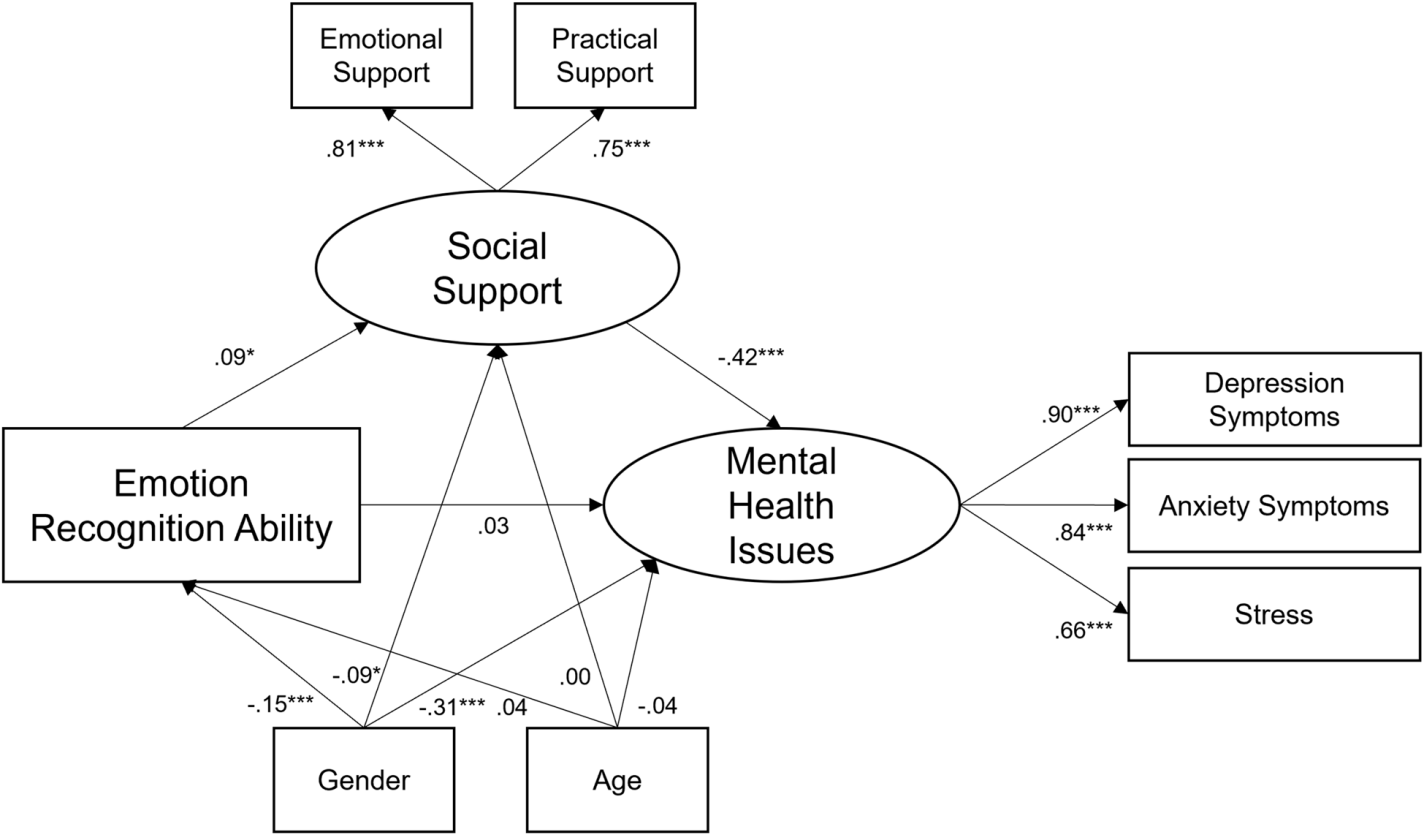
Longitudinal mediation effect of social support (T2) on the link between emotion recognition ability (T1) and mental health issues (T2). Total Effect of ERA on mental health issues = −0.01 (*z* = −0.21, *p* = 0.833). Monte Carlo test for Mediation^54^: Indirect effect = 0.04 (*z* = −2.30, *p* = 0.019). Model Fit: CFI = 0.994, RMSEA = 0.031, SRMR = 0.018, Chi2 = 25.08 (*p* = 0.023). All coefficients are standardized. *N* = 986. *p < 0.05. ** *p* < 0.01. *** *p* < 0.001.

Bivariate correlations and descriptive statistics of the variables of interest are displayed in Tables 2 and 3. Figure 1 depicts the structural equation model of the mediation effect of ERA on mental health issues through perceived social support. The model demonstrated a good fit. There was no significant total effect of ERA on mental health issues; however, the indirect effect via perceived social support was significant with a small effect size. The direct effect of ERA shifted from negative to positive with the inclusion of the mediation path but remained non-significant. This means that although higher ERA did not directly predict lower mental health issues one year later, it increased perceived social support which in turn decreased mental health issues, suggesting a “hidden path” through which ERA affected mental health.

**Table 3:** Descriptive statistics.

Variables	Time point	M	SD	Min	Max	Cronbach’s alpha	Skewness	Kurtosis
Emotion recognition ability	T1	0.71	0.09	0.43	0.95	0.53	−0.27	2.95
Emotional support	T2	8.59	1.97	0	10	NA	−1.84	6.44
Practical support	T2	7.72	2.31	0	10	NA	−1.16	3.92
Depression symptoms	T2	18.48	11.05	0	58	0.92	0.69	3.02
Anxiety symptoms	T2	44.67	11.72	20	80	0.93	0.11	2.48
Stress	T2	5.41	2.19	1	10	NA	−0.19	1.95
Burnout: emotional exhaustion	T2	16.76	5.07	5	30	0.87	0.17	2.82
Burnout: cynicism	T2	9.74	4.48	4	24	0.86	0.86	3.27
Burnout: academic efficacy	T2	24.13	4.55	6	36	0.77	−0.14	2.94

**Fig. 2:**
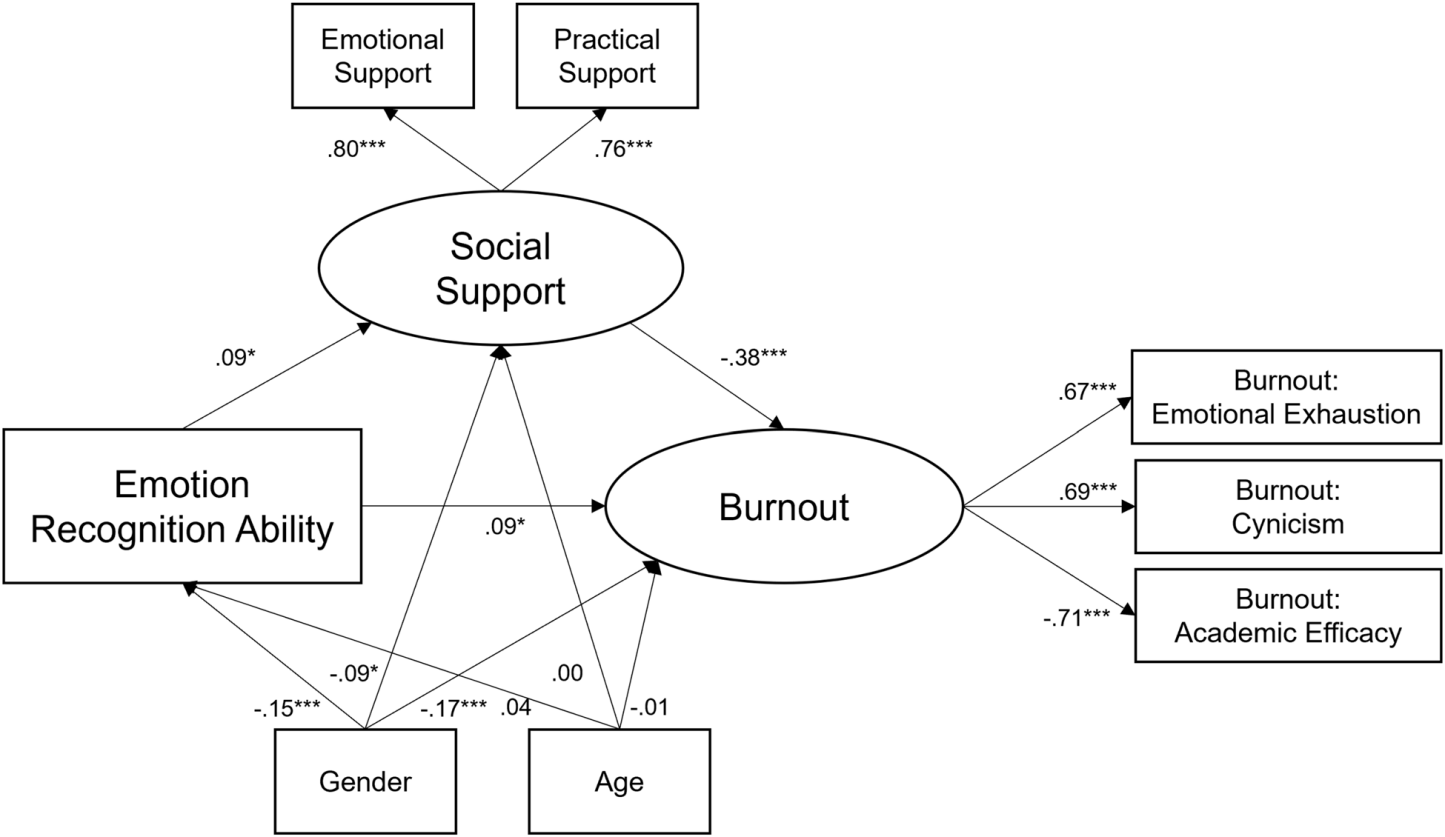
Longitudinal mediation effect of social support (T2) on the link between emotion recognition ability (T1) and burnout (T2). Total Effect of ERA on mental health issues = 0.05 (*z* = 1.34, *p* = 0.182). Monte Carlo Test for Mediation^54^: Indirect effect = −0.04 (*z* = −2.17, *p* = 0.030). Model Fit: CFI = 0.952, RMSEA = 0.069, SRMR = 0.033, Chi2 = 73.27 (*p* < 0.001). All coefficients are standardized. *N* = 986. *p < 0.05. **p < 0.01. ***p < 0.001.

Figure 2 shows the structural equation model of the mediation effect of ERA on burnout through perceived social support. The model demonstrated a good fit, except for the RMSEA, which was close to the recommended threshold of 0.06^53^. Again, there was no significant total effect of ERA on burnout but the indirect effect via perceived social support was significant. Interestingly, when accounting for the mediation, the direct effect of ERA was positive and significant, indicating a suppression effect: When the mediation path through social support was considered, ERA predicted higher burnout.

Taken together, H1 was rejected: ERA did not directly predict lower mental health issues or burnout overall one year later. However, H2 was supported: ERA predicted increased social support (H2a), which in turn predicted lower mental health issues and burnout (H2b), with significant indirect effects. Additionally, when accounting for this mediation path, the direct effects of ERA on mental health issues and burnout became more positive (and significant for burnout). This means that, aside from social support, there may be another factor at play through which ERA increases mental health issues and that cancels out the positive path through social support.

### Exploratory analyses

Exploratory analyses were conducted to examine possible negative pathways between ERA and mental health. One possibility is that higher ERA involves a stronger reactivity to negative emotional situations, which can adversely affect mental health. Previous studies have found that individuals with higher ERA were more reactive to public speaking stress^31^, appraised negative events as more relevant and negative^30^, and reported a higher tendency for ruminative emotion regulation^25^. Therefore, a secondary mediation path via coping mechanisms was explored using items from the coping section of the Euronet questionnaire, which measures habitual reactions to difficulties^15,56^. Due to the debatable factor structure and reliability of the original scale^56^, three items were selected from the T2 assessment to represent different coping mechanisms: (1) problem-focused (“I analyze the situation and try to solve/overcome the problem”), (2) emotion-focused (“I cry”), and (3) avoidance coping (“I withdraw and hide”). These items were rated on a scale from 0 (“not at all usual for me”) to 3 (“very usual for me”). Descriptives and bivariate correlations with the other variables are provided in Supplementary Tables S1 and S2.

**Fig. 3:**
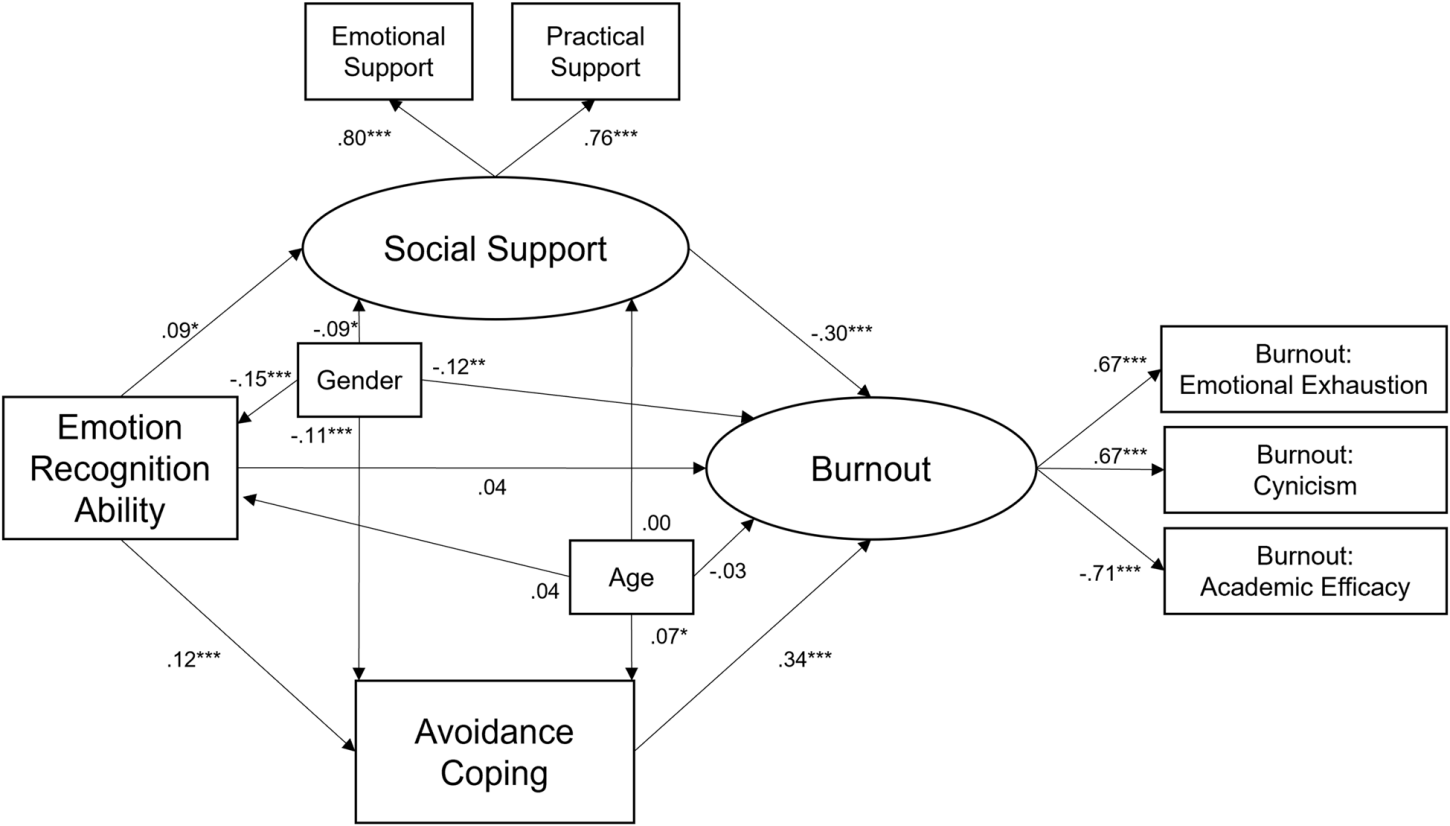
Longitudinal double-mediation effect of social support (T2) and avoidance coping (T2) on the link between emotion recognition ability (T1) and burnout (T2). Total effect of ERA on burnout = 0.05 (*z* = 1.31, *p* = 0.189). Monte Carlo tests for mediation^54^: Indirect effect via social support = −0.03 (*z* = −2.03, *p* = 0.042); Indirect effect via avoidance coping = .04 (*z* = 3.41, *p* = 0.001). Model Fit: CFI = 0.915, RMSEA = .086, SRMR = .058, Chi2 = 139.47 (p < 0.001). All coefficients are standardized. *N* = 986. *p < 0.05. **p < 0.01. ***p < 0.001.

In a series of structural equation models, each coping item was added separately as a secondary mediator to the two models predicting mental health issues and burnout shown in Figures 1 and 2. As an example, Figure 3 shows the secondary mediation of avoidance-coping on burnout (see Supplementary Figures S1 to S5 for all other models). Results showed that increased avoidance coping mediated the effect of ERA on both mental health issues (Figure S5) and burnout (Figure 3). The mediation effects via problem-focused and emotion-focused coping on mental health issues and burnout were non-significant (Figures S1 to S4). All models showed acceptable to good fit (CFI: 0.915–0.990; RMSEA: 0.039–0.086; SRMR: 0.021–0.058). Including the mediation path via avoidance coping alongside the path via social support reduced the direct effects of ERA on mental health issues and burnout. This indicates that ERA had both beneficial (via higher social support) and detrimental indirect effects (via higher avoidance in response to difficulties) on mental health and burnout.

In another exploratory analysis we investigated whether the longitudinal analyses may have suffered from attrition bias due to more severe mental health issues in the dropout sample. To achieve this, we compared the mental health and burnout scores of the included sample at T1 with the dropout sample, i.e., participants that did not participate in two consecutive years. Of the latter, we further excluded participants that were in their sixth curriculum year at first participation and therefore were not able to participate a second time, resulting in a dropout sample of *n* = 734. Welch’s t-tests indicated that the dropout sample reported higher depression, *t*(1444) = 5.25, *p* < 0.001, *d* = 0.26, anxiety, *t*(1487) = 4.46, *p* < 0.001, *d* = 0.22, and stress, *t*(1558) = 3.12, *p* = 0.002, *d* = 0.15, as well as lower academic efficacy, *t*(1496) = -4.09, *p* < 0.001, *d* = -0.20, than the analysis sample at T1, with small effect sizes. The included and dropout samples did not differ significantly in emotional exhaustion, *t*(1515) = 0.25, *p* = 0.800, *d* = 0.01, and cynicism, *t*(1422) = 1.69, *p* = 0.092, *d* = 0.08. This implies that the results found in the present study may not be applicable to the students that were most strongly afflicted by mental health issues. The full statistics for these analyses are presented in Supplementary Table S3 in the supplementary materials.

## Discussion

The present study investigated whether and how the ability to accurately judge other people’s emotions contributes to better intrapersonal functioning by reducing mental health issues such as stress, depression, anxiety, and burnout in a large sample of medical students. These findings are particularly relevant in light of their high prevalence of mental health issues and burnout, which may extend into their future healthcare practice. The first notable finding was in line with previous studies in which ERA failed to show direct effects on well-being in non-clinical samples^25^: Medical students with higher ERA scores did not report fewer mental health issues and lower burnout one year later. Given the longitudinal design, large sample, and multifaceted measurement of mental health issues in the present study, this finding adds to the evidence that there is no direct benefit of ERA to mental health and subjective well-being^12,25^. However, the results also showed that although a direct link was missing, higher ERA increased the perceived availability of social support, which in turn reduced mental health issues. Additionally, exploratory analyses revealed that ERA was linked to more avoidance coping in difficult situations, which in turn increased mental health issues. Taken together, these results highlight the complex pathways through which ERA may be linked to mental health in future healthcare professionals.

The finding that ERA was indirectly linked to mental health issues and burnout through perceived social support aligns with the notion that accurately interpreting other people’s emotions helps in building meaningful, reliable, and stable relationships, or generally, high relationship quality^1^. According to theories on ability EI^7^ and interpersonal accuracy^6^, ERA facilitates the adaptive use of other socio-emotional competences in social interactions and relationships (e.g., managing others’ emotions to increase cooperation and trust). This results in greater availability of practical and emotional social support when needed and may help high ERA individuals to better manage stressful life situations and prevent declines in mental health. The beneficial interpersonal pathway between ERA and mental health, found despite the absence of an overall link, suggests that similar pathways may have been at play in previous studies with overall null findings, which typically only tested the direct link^25^. Taken together, these results suggest that ERA may indirectly support healthcare professionals’ mental health through enhanced interpersonal resources, potentially mitigating the effects of high-pressure work environments.

The “hidden” positive effect of ERA suggests that ERA may simultaneously impact mental health through negative pathways that cancel each other out. Our exploratory analyses revealed another “hidden” path: High ERA predicted more avoidance coping, i.e., the tendency to react to difficulties with withdrawal, which correlated with higher self-reported burnout symptoms. Previous studies have linked ERA, particularly the GERT used here, to higher neuroticism and rumination^25,57^. Furthermore, individuals with higher ERA tend to perceive negative events as more severe, relevant, and difficult to manage^30^. Schlegel^12^ suggested that individuals with high ERA may be more attuned to negative emotions around them, especially in stressful situations. Medical students, who regularly encounter stress in academic and clinical settings, might be particularly sensitive to negative emotional cues in others. Taken together, ERA appears to have both positive and negative impacts on medical students’ mental health, through interpersonal (e.g., social support) and intrapersonal (e.g., emotional reactivity) pathways. Such “bright” and “dark” sides have been previously hypothesized for both overall EI^27^ and ERA specifically^12^. The ability to recognize emotional expressions has been shown to increase through training^58^, therefore it is crucial that such training also focuses on using ERA adaptively to foster beneficial pathways and avoid detrimental effects, for instance, by combining ERA training with emotion regulation training. However, no studies are known to have investigated effects of ERA training on mental health, burnout, or the intermediary outcomes social support and coping in medical students or health professionals, and long-term effects are unclear^58^.

The present study has several strengths, but also some limitations. A clear strength is the longitudinal design, which expands previous research and indicates temporal effects of ERA. Nevertheless, the presented analyses cannot attest causality to the effects that were found. Future studies should therefore study more timepoints or examine the effects of training interventions. Additionally, the large sample of medical students made it possible to detect small effects in a sample at risk of developing mental health issues. It remains an open question whether these findings generalize to other groups of healthcare professionals (e.g., to nurses or psychotherapists) or broader samples (e.g., other age groups and professions). Also, the reported focal indirect and total effects of ERA on mental health showed small effect sizes, which may limit practical implications. Nevertheless, small effect sizes when predicting real life outcomes are common in EI and interpersonal accuracy research^1^. Such small effects may cumulate over time and situations, and may combine with other effects to meaningfully contribute to social support, coping, mental health and burnout in health professionals^59,60^. Furthermore, exploratory analyses showed a possible attrition bias due to more severe mental health issues. Although the found differences between included and dropout samples were small, they might imply that our results do not apply in the same way to students presenting severe mental health issues.

A further limitation is the relatively low internal consistency of the ERA measure (GERT-S) in the present study, though it has demonstrated good psychometric properties in prior research^43,57,61^. One potential explanation is the homogeneity of the medical student sample, which may have reduced variance and measurement reliability, possibly leading to an underestimation of effect sizes^62^. Additionally, social support was not a specific focus of the original ETMED-L project^15^, which is why the present study was limited to the investigation of social support with a measure consisting of only two items. This limited the level of detail in assessing the social support medical students needed and received, but this two-item scale has been successfully applied in previous panel studies^46^ and longitudinal physical and mental health research^14,63^. Nevertheless, future studies should investigate social support with more detailed measures such as the Multidimensional Scale of Perceived Social Support^64^. Furthermore, the exploratory mediation analyses on coping presented in this study require replication, and future studies require a more comprehensive assessment of coping. A more general limitation is the reliance on self-report questionnaires which are prone to socially desirable responding, recall bias, and other issues, except for ERA, which was measured with a performance-based test. Lastly, the analyses in the present study did not control confounders beyond gender and age, such as the students’ academic workload, socioeconomic status, or prior history of mental health issues, which could have influenced the measured mental health issues.

To conclude, the present study showed that being more accurate at reading others does not directly impact mental health. However, the study demonstrated that ERA can simultaneously improve mental health in medical students through interpersonal processes and impair it through intrapersonal pathways. These findings imply that future studies in the domains of ERA, ability EI, and interpersonal accuracy in healthcare workers should go beyond the examination of direct links with well-being and consider the underlying mechanisms in much more detail. To this end, future research should develop a comprehensive theoretical framework to map these intrapersonal and interpersonal pathways and further explore ERA’s potential in supporting resilience among healthcare professionals.

## Supplementary Information

Supplementary Information.

## Data Availability

The data that support the findings of this study are openly available on zenodo at http://doi.org/10.5281/zenodo.12517444.
