# Supplementary material for: Social support and avoidance mediate positive and negative effects of emotion recognition ability on mental health in medical students
Source: Sci Rep. 2025 May 15;15:16910. doi: 10.1038/s41598-025-02025-8 (PMC12081610; doi:10.1038/s41598-025-02025-8)
Supplement: Supplementary file 1 — Supplementary Information. [file 41598_2025_2025_MOESM1_ESM.pdf]

Supplementary Materials

Supplementary materials to the research article "Social support and avoidance explain positive and negative effects of emotion recognition ability on mental health in medical students" by Nils R. Sommer, Valerie Carrard, Céline Bourquin, Alexandre Berney, Katja Schlegel.

Supplementary Tables and Figures

Variables	Time point	M	SD	Skewness	Kurtosis
**(12) Problem-Focused Coping**	T2	2.29	0.65	-0.60	3.32
**(13) Emotion-Focused Coping**	T2	1.33	1.08	0.14	1.72
**(14) Avoidance Coping**	T2	0.93	0.97	0.61	2.19

Table S1. Descriptive Statistics of reactions to difficulties items.

Variables	(1)	(2)	(3)	(4)	(5)	(6)	(7)	(8)	(9)	(10)	(11)	(12)	(13)
**(1) Emotion Recognition Ability**	1.00												
**(2) Emotional Support**	0.12	1.00											
**(3) Practical Support**	0.04	0.61	1.00										
**(4) Depression Symptoms**	0.01	-0.29	-0.29	1.00									
**(5) Anxiety Symptoms**	0.05	-0.24	-0.25	0.75	1.00								
**(6) Stress**	0.03	-0.15	-0.14	0.60	0.56	1.00							
**(7) Burnout: Emotional Exhaustion**	0.03	-0.18	-0.20	0.62	0.57	0.51	1.00						
**(8) Burnout: Cynicism**	0.07	-0.17	-0.15	0.44	0.35	0.23	0.47	1.00					
**(9) Burnout: Academic Efficacy**	-0.04	0.21	0.25	-0.47	-0.48	-0.32	-0.46	-0.50	1.00				
**(10) Gender Identification**	-0.15	-0.09	-0.07	-0.23	-0.26	-0.19	-0.19	-0.06	0.05	1.00			
**(11) Age**	0.03	0.01	-0.01	-0.07	-0.04	-0.05	-0.10	0.07	0.01	0.08	1.00		
**(12) Problem-Focused Coping**	0.04	0.12	0.11	-0.22	-0.33	-0.18	-0.18	-0.13	0.28	0.14	0.10	1.00	
**(13) Emotion-Focused Coping**	0.13	0.10	0.06	0.28	0.35	0.21	0.24	0.13	-0.11	-0.58	-0.07	-0.22	1.00
**(14) Avoidance Coping**	0.14	-0.20	-0.19	0.46	0.42	0.27	0.29	0.24	-0.31	-0.13	0.06	-0.16	0.15

Table S2. Extended bivariate correlations including reactions to difficulties. Gender Identification: Female / nonbinary = 0, male = 1.

**Variables**	**Analysis Sample (n = 986) at T1**		**Dropout Sample (n = 734)**				
	**M**	**SD**	**M**	**SD**	**t_{Welch} (df)**	**p**	**d**
**Depression Symptoms**	18.60	10.73	21.56	12.00	5.25 (1444)	<.001	0.26
**Anxiety Symptoms**	44.08	11.63	46.72	12.45	4.46 (1487)	<.001	0.22
**Stress**	5.51	2.14	5.84	2.12	3.12 (1558)	.002	0.15
**Burnout: Emotional Exhaustion**	17.05	4.94	17.11	5.13	0.25 (1515)	.800	0.01
**Burnout: Cynicism**	9.31	4.12	9.69	4.71	1.69 (1422)	.092	0.08
**Burnout: Academic Efficacy**	24.54	4.42	23.63	4.69	-4.09 (1496)	<.001	-0.20

Table S3. Comparison of mental health and burnout scores at first assessment between analysis sample and dropout sample.

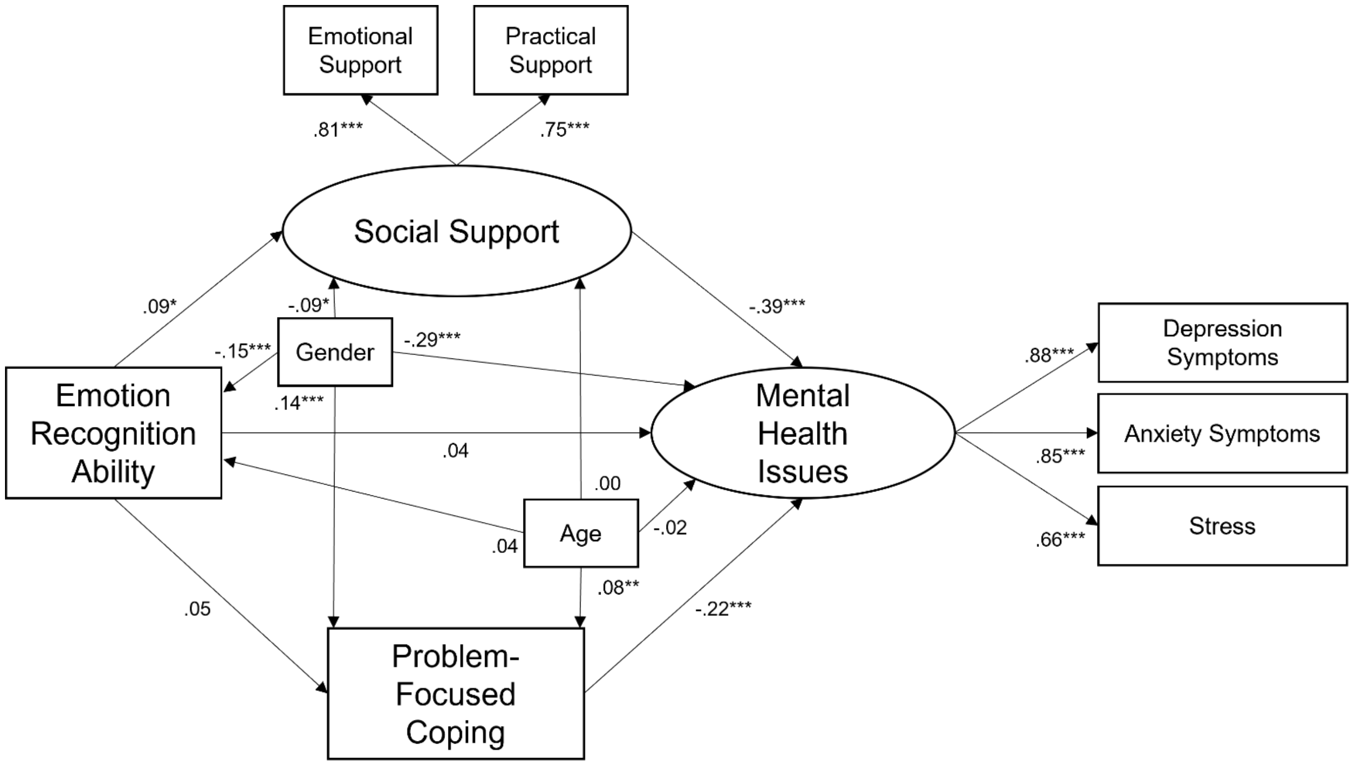

Figure S1. Longitudinal double-mediation effect of social support (T2) and problem-focused coping (T2) on the link between emotion recognition ability (T1) and mental health issues (T2). Total Effect of ERA on mental health issues = -.005 (z = -0.14, p = .887). Monte Carlo Tests for Mediation[1]: Indirect effect via social support = -.04 (z = -2.34, p = .020); Indirect effect via problem-focused coping = -.01 (z = 0.01, p = .093). Model Fit: CFI = .971, RMSEA = .060, SRMR = .035, Chi2 = 77.84 (p <.001). All coefficients are standardized. N = 986. * p < .05. ** p < .01. *** p < .001.

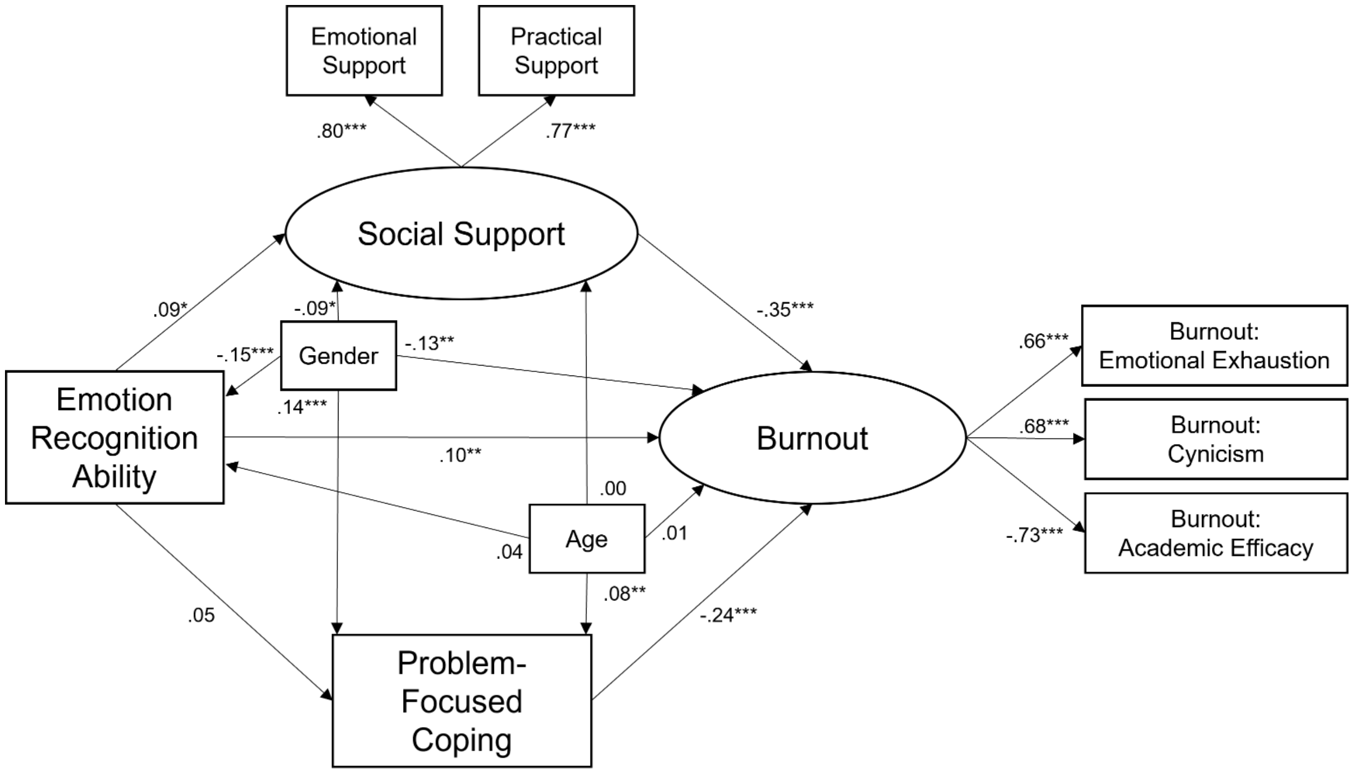

Figure S2. Longitudinal double-mediation effect of social support (T2) and problem-focused coping (T2) on the link between emotion recognition ability (T1) and burnout (T2). Total Effect of ERA on burnout = .05 (z = 1.33, p = .183). Monte Carlo Tests for Mediation[1]: Indirect effect via social support = -.03 (z = -2.12, p = .034); Indirect effect via problem-focused coping = -.01 (z = -1.68, p = .094). Model Fit: CFI = .930, RMSEA = .076, SRMR = .043, Chi2 = 113.376 (p < .001). All coefficients are standardized. N = 986. * p < .05. ** p < .01. *** p < .001.

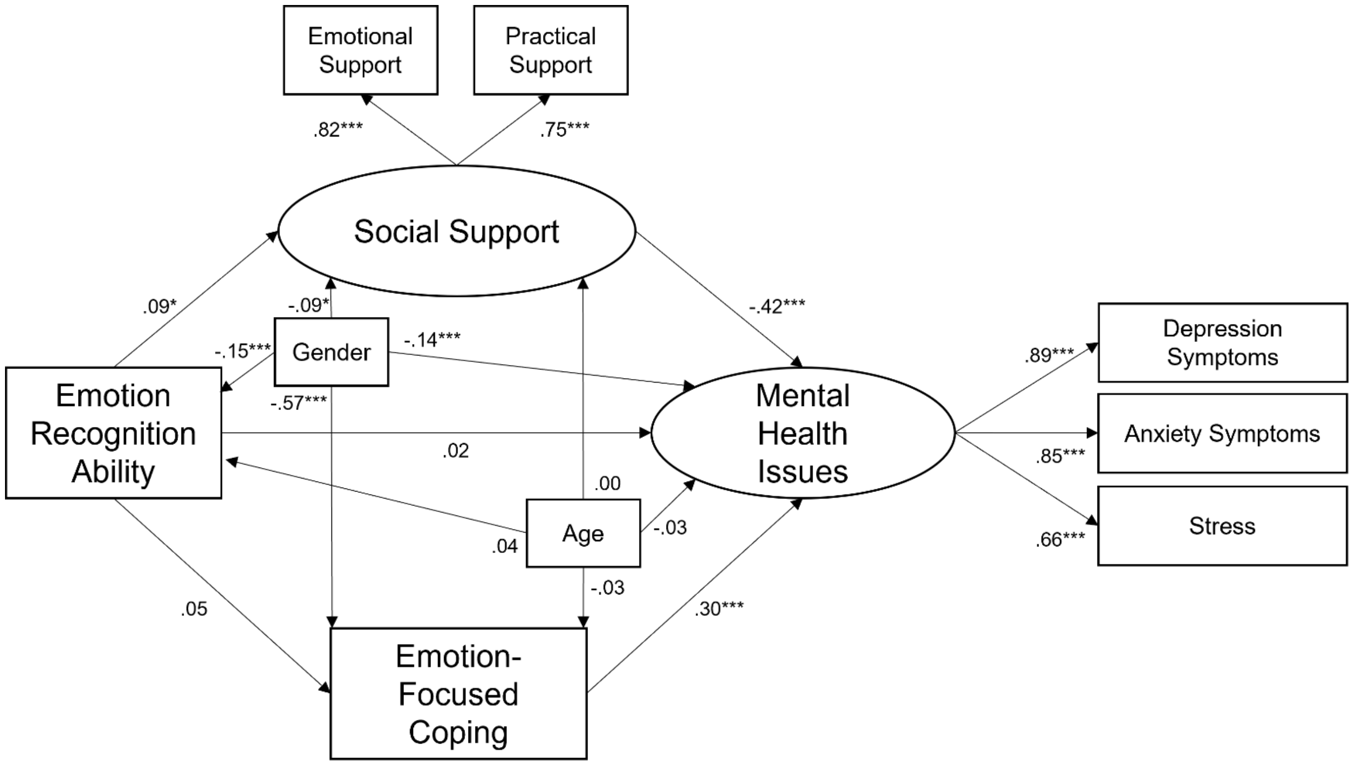

Figure S3. Longitudinal double-mediation effect of social support (T2) and emotion-focused coping (T2) on the link between emotion recognition ability (T1) and mental health issues (T2). Total Effect of ERA on mental health issues = -.006 (*z* = -0.17, *p* = .867). Monte Carlo Tests for Mediation[1]: Indirect effect via social support = -.04 (*z* = -2.43, *p* = .015); Indirect effect via emotion-focused coping = .02 (*z* = 1.73, *p* = .084). Model Fit: CFI = .990, RMSEA = .039, SRMR = .021, Chi2 = 42.542 (p = .001). All coefficients are standardized. *N* = 986. * *p* < .05. ** *p* < .01. *** *p* < .001.

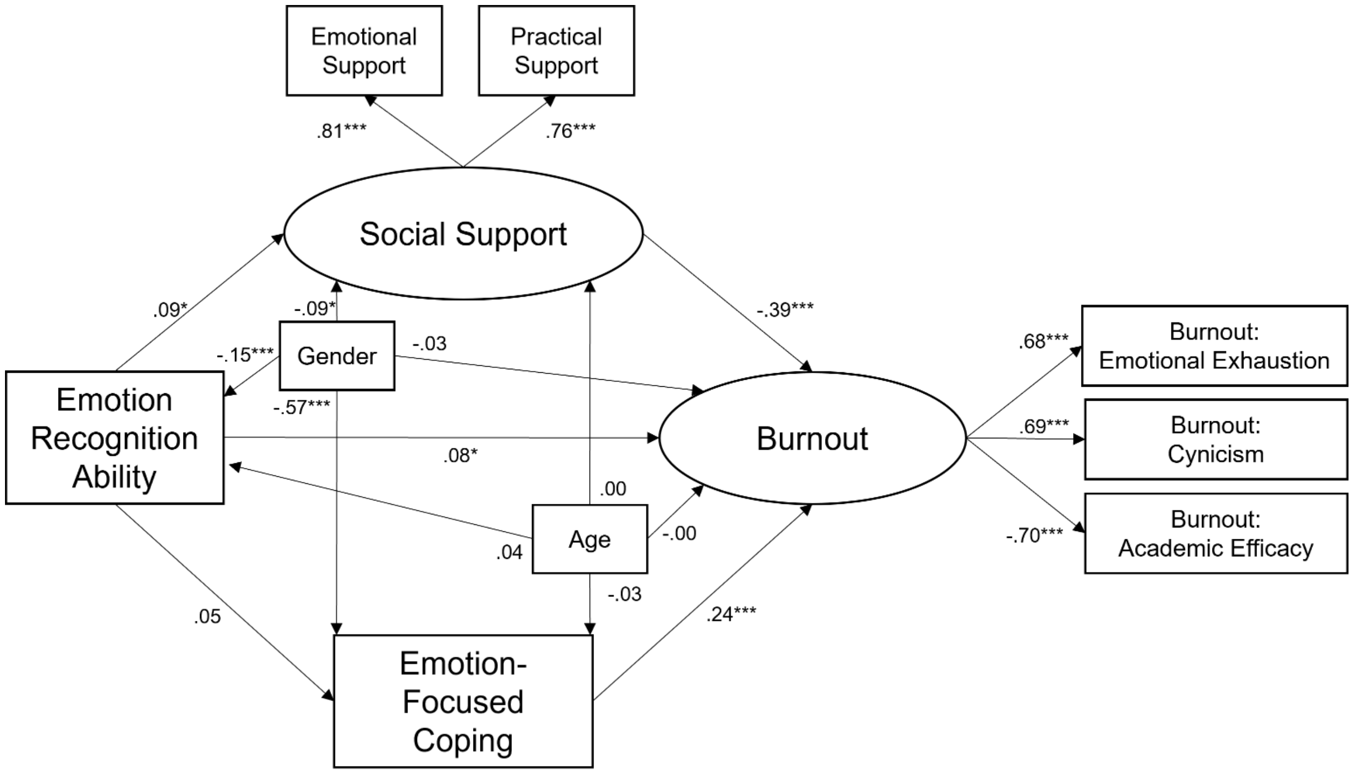

Figure S4. Longitudinal double-mediation effect of social support (T2) and emotion-focused coping (T2) on the link between emotion recognition ability (T1) and burnout (T2). Total Effect of ERA on burnout = .05 (z = 1.32, p = .187). Monte Carlo Tests for Mediation[1]: Indirect effect via social support = -.04 (z = -2.25, p = .025); Indirect effect via emotion-focused coping = .01 (z = 1.67, p = .095). Model Fit: CFI = .963, RMSEA = .062, SRMR = .034, Chi2 = 80.95 (p < .001). All coefficients are standardized. N = 986. * p < .05. ** p < .01. *** p < .001.

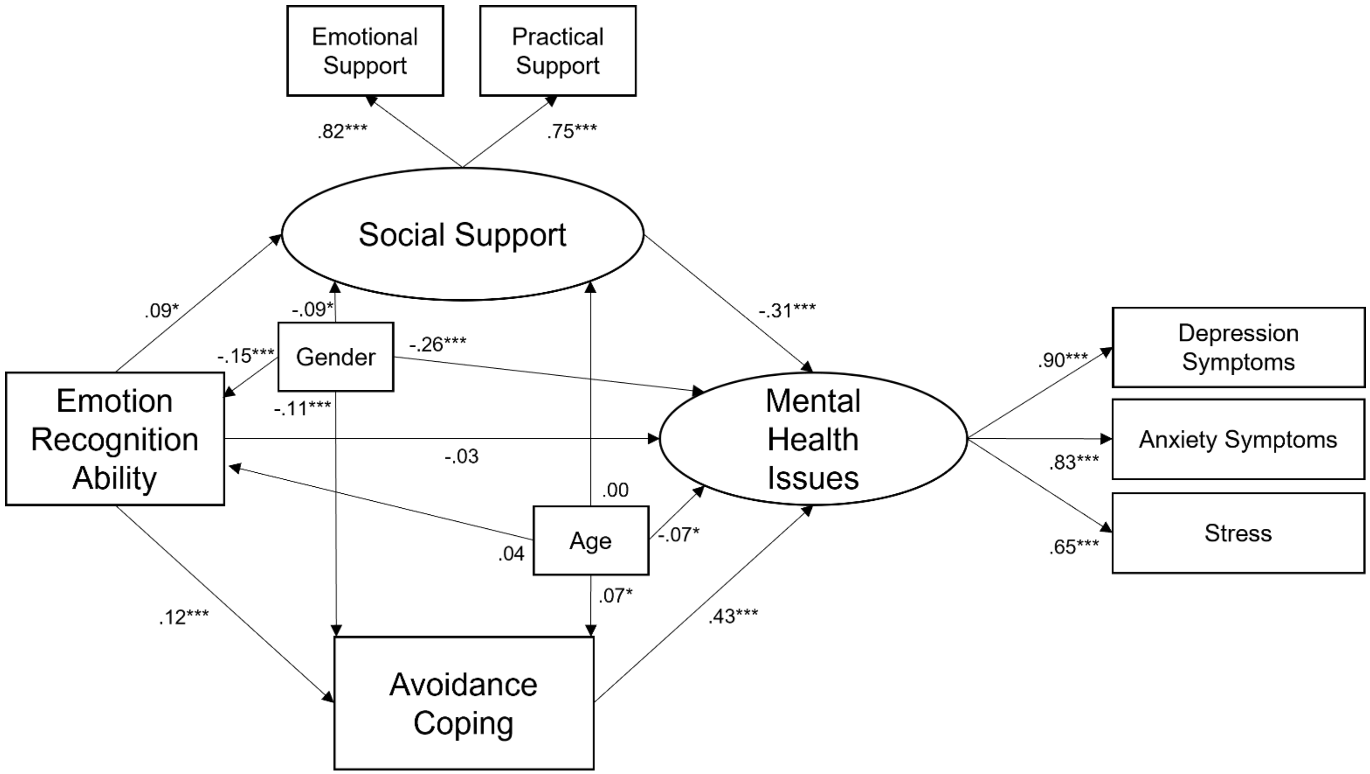

Figure S5. Longitudinal double-mediation effect of social support (T2) and avoidance coping (T2) on the link between emotion recognition ability (T1) and mental health issues (T2). Total Effect of ERA on mental health issues = -.01 (z = -0.24, p = .811). Monte Carlo Tests for Mediation[1]: Indirect effect via social support = -.03 (z = -2.27, p = .023); Indirect effect via avoidance coping = .05 (z = 3.60, p < .001). Model Fit: CFI = .967, RMSEA = .067, SRMR = .056, Chi2 = 93.02 (p < .001). All coefficients are standardized. N = 986. * p < .05. ** p < .01. *** p < .001.

References

1. Zhao, X., Lynch, J. G., Jr. & Chen, Q. Reconsidering Baron and Kenny: Myths and Truths about Mediation Analysis. *J. Consum. Res.* **37**, 197–206 (2010).
